# Perspectives on the design and methodology of periconceptional nutrient supplementation trials

**DOI:** 10.1186/s13063-015-1124-0

**Published:** 2016-01-30

**Authors:** Bernard J. Brabin, Sabine Gies, Stephen Owens, Yves Claeys, Umberto D’Alessandro, Halidou Tinto, Loretta Brabin

**Affiliations:** Clinical Division, Liverpool School of Tropical Medicine, Pembroke Place, Liverpool, L35QA UK; Global Child Health Group, Academic Medical Centre, University of Amsterdam, Amsterdam, The Netherlands; Department of Biomedical Sciences, Institute of Tropical Medicine, Antwerp, Belgium; Northumbria Healthcare NHS Foundation Trust, North Shields, NE29 8NH UK; Clinical Sciences Department, Institute of Tropical Medicine, Antwerp, Belgium; Medical Research Council Unit (MRC), Fajara, The Gambia; London School of Hygiene and Tropical Medicine, London, UK; Institute of Tropical Medicine, Antwerp, Belgium; Clinical Research Unit of Nanoro (URCN/IRSS), Nanoro, Burkina Faso; Institute of Cancer Sciences, University of Manchester, Manchester, UK

**Keywords:** Periconceptional, Pregnancy, Placenta, Iron, Folic acid, Micronutrients, Adherence

## Abstract

Periconceptional supplementation could extend the period over which maternal and fetal nutrition is improved, but there are many challenges facing early-life intervention studies. Periconceptional trials differ from pregnancy supplementation trials, not only because of the very early or pre-gestational timing of nutrient exposure but also because they generate subsidiary information on participants who remain non-pregnant. The methodological challenges are more complex although, if well designed, they provide opportunities to evaluate concurrent hypotheses related to the health of non-pregnant women, especially nulliparous adolescents. This review examines the framework of published and ongoing randomised trial designs. Four cohorts typically arise from the periconceptional trial design — two of which are non-pregnant and two are pregnant — and this structure provides assessment options related to pre-pregnant, maternal, pregnancy and fetal outcomes. Conceptually the initial decision for single or micronutrient intervention is central — as is the choice of dosage and content — in order to establish a comparative framework across trials, improve standardisation, and facilitate interpretation of mechanistic hypotheses. Other trial features considered in the review include: measurement options for baseline and outcome assessments; adherence to long-term supplementation; sample size considerations in relation to duration of nutrient supplementation; cohort size for non-pregnant and pregnant cohorts as the latter is influenced by parity selection; integrating qualitative studies and data management issues. Emphasis is given to low resource settings where high infection rates and the possibility of nutrient-infection interactions may require appropriate safety monitoring. The focus is on pragmatic issues that may help investigators planning a periconceptional trial.

## Background

Trials of periconceptional folic acid supplementation to reduce neural tube defects [1, 2] and of iodised oil administered early in pregnancy to avoid cretinism [3] identified the major influence on clinical outcomes of maternal nutritional status during the first trimester of pregnancy. Since these early studies, many trials have been conducted, mostly with second and third trimester nutrient supplementation [4]. The majority have compared iron plus folic acid supplementation with interventions using variable compositions of micronutrients and vitamins. Meta-analyses and systematic reviews of clinical outcomes have reported varied results, from null effects on neonatal mortality [5] to improvement in general health indicators such as birth weight. Most showed no differences in the risk of pre-term birth, stillbirth, maternal or neonatal outcomes [6], with conflicting results for infant mortality reductions [7]. Null effects [8] or varied findings were reported for fetal growth restriction [8, 9]. Importantly, an increased risk of neonatal death was reported with multimicronutrient compared with iron/folate interventions after the first trimester of pregnancy [6, 10]. The evidence to support daily iron and folate supplementation in pregnancy is based mainly on its beneficial effects on maternal anaemia [11, 12], with micronutrients providing no additional benefit on third trimester maternal anaemia compared with iron-folic acid alone [9, 13].

None of the above trials started with mechanistic hypotheses. Improved understanding of nutritional intermediate pathways could explain differences between trials in fetal and pregnancy outcomes, but this requires integrated placental and biomarker studies and infection profiling from early in gestation [14]. Critical nutritional periods in early pregnancy include the pre-embryonic and embryonic developmental stages. If a nutrient exposure during these periods is associated either positively or negatively with the postulated clinical outcome, a causal pathway is implicated. Periconceptional trials re-focus attention on the fetal growth effects of placental vascularisation and function and the mechanisms determining fetal and placental phenotypes [15, 16]. Recently described effects of maternal overweight on cardiometabolic disease risk in the offspring on later adult disease indicate that early gestational nutritional influences can have lifelong effects [17]. Given that the periconceptional use of nutrition supplements has been assessed in few controlled trials [18], their mechanistic basis is even less evident.

In view of the potential importance of pre-pregnancy supplementation, this paper outlines several aspects related to the framework of periconceptional supplementation trials, including: underlying mechanistic hypotheses; single and multimicronutrient supplementation as alternative trial interventions; sample size considerations in relation to duration of nutrient supplementation, cohort size and composition; outcome measurement options and characteristics; design options, with comparison of published and current study designs, outcome assessments and supplement adherence; the role of qualitative studies within trial designs and data management options. Without improved insights into the nature and conduct of these studies there is a risk that they will yield confusing results. Statistical issues specific to data analysis are not included in the review, as these are trial specific.

The main emphasis is on developing countries where nutritional deficiencies are common and adolescents give birth while still growing [19]. Trying to improve nutrition before the first pregnancy is attractive, especially if this helps to optimise nutrient requirements throughout pregnancy.

## Review

### Methods

**Fig. 1:**
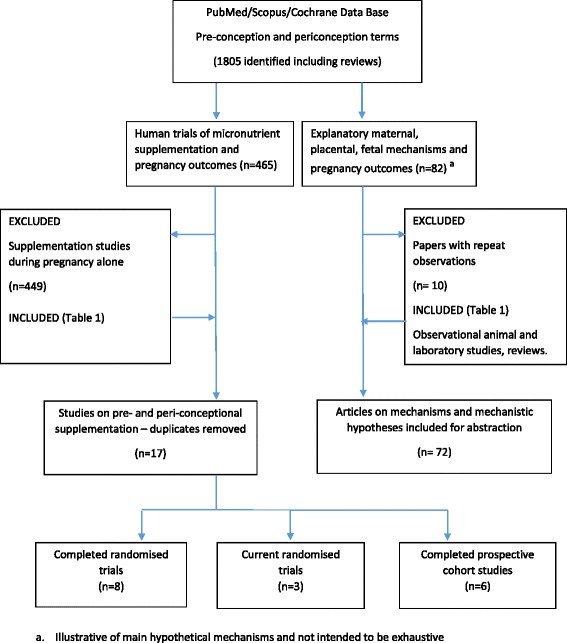
PRISMA flow diagram

We identified published studies using PubMed and Scopus search engines and the Cochrane Central Register of Controlled Trials (CENTRAL; The Cochrane Library). Search terms included micronutrient supplements, periconceptional period, placentation, embryogenesis, pregnancy outcomes, birth weight, growth restriction, iron, folic acid, and micronutrients. For identification of randomised controlled trials or prospective cohort studies we identified human studies on periconceptional interventions with nutrition supplements covering the period 1950 to July 2015 with no language restriction. Information from recent meta-analyses and systematic reviews addressing the periconceptional period, pre-conception, or pre-pregnancy nutrient supplement interventions was reviewed. Ongoing randomised trials with published or available methodologies but still awaiting trial closure were included. Observational studies were excluded, unless population based, as these may be confounded by baseline differences in the prevalence of one or more nutrient deficiencies. Figure 1 shows the PRISMA flow diagram, and Table 1 lists the study inclusion and exclusion parameters. Animal studies to identify biological mechanisms for nutritional regulation of fetal growth and gestational length were reviewed to identify mechanistic hypotheses.

**Table 1:** Study inclusion and exclusion parameters

Study inclusion parameters	Study exclusion parameters
- Human studies from 1950 to July 2015, with no language restriction	- Studies with nutrient interventions commencing during, but not before pregnancy
- Blinded and unblinded randomised trials	- If time of intervention was unclear
- Community-/population-based studies	- Poorly defined control or comparison groups
- Ongoing randomised trials studies with published methodologies, but awaiting trial closure	- Observational human studies
- Clear definition of nutrient intervention	
- Indication of period of periconceptional supplementation	
- Definition of outcome variables (maternal, fetal, or infant outcomes).	
- Studies in non-pregnant women prior to ascertainment of pregnancy	
- Starting early in first trimester up to 28 days after last menstrual period	
- Individually randomised or population-based studies	

As definition of the periconceptional period varied across studies, here it has been defined as taking supplements before, or at the time of, the last menstrual period prior to conception, and up until the end of the first trimester. Overall quality of evidence for outcomes was not graded, as the purpose of this review was to assess methodological frameworks, and not the quality of the evidence for a particular outcome. For the majority of cohort studies published, grading schemes for quality of evidence had previously been applied [6].

## Results and discussion

### Trial structures and outcome assessments

Figure 2 illustrates a schema for 17 periconceptional studies identified for this review. Nine studies were double blind randomised trials [20–28], two unblinded randomised trials [29, 30], and six community- or population-based unblinded prospective studies [29, 31–34, 36]. Six randomised trials had two cohorts [20, 21, 24, 25, 30, 31], and five trials three or four cohorts [22, 23, 26, 28, 29]. Three included untreated controls [22, 23, 29], and four received placebo [20, 25, 27, 32] and the remainder a control intervention [21, 24, 26, 30]. All the community- or population-based studies defined two study arms [33–37], with one using four study arms [38]. Five included untreated control groups [34–38]. They covered 24 countries with the earliest randomised trial reported in 1981 [20]. The study sample sizes in Fig. 2 refer to numbers at enrolment. Studies with two intervention arms in single country locations are listed first, followed by trials with three or four intervention arms or with multiple country locations. Three were in sub-Saharan Africa [21, 29, 31], five in Europe [20, 22, 23, 30, 37], eight in Asia [24–26, 28, 29, 33–35], with single locations in Cuba [36], Algeria [38], Guatemala, Australia, Canada, Israel and Russia [23]. The minimum period of pre-pregnancy supplementation was 28 days or one month, and the maximum 3.5 years, with the majority providing pre-pregnancy supplementation for a period less than nine months. All studies supplemented through the first trimester; one randomised trial continued supplementation in one arm through later trimesters [29], and another trial continued supplementation after an intervening pregnancy [28].

**Fig. 2:**
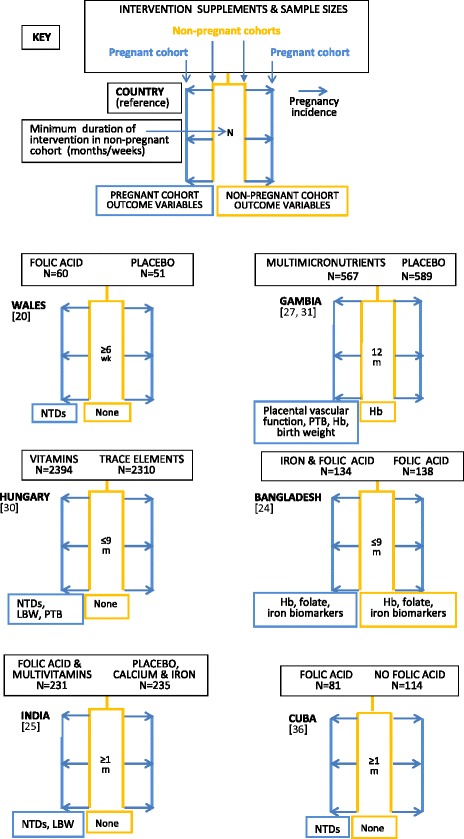
Summary outlines of periconceptional nutrition supplement intervention studies. Description of intervention supplements uses investigators’ terminology. Multimicronutrient contents of intervention supplements summarised in Table 3. All intervention regimens are daily unless specified. Regimen details, blinding and duration are outlined in Table 8. Hb: haemoglobin; NTDs: neural tube defects; IDA: iron deficiency anaemia; PTB: pre-term birth; LBW: low birth weight; SB: stillbirth; HC: head circumference; Brackets: reference number

For non-pregnant cohorts in the randomised trials, that is, study subjects who did not become pregnant during the study period, six trials reported no outcome measurements and no end assessment survey [20, 22, 23, 25, 26, 30], five reported haemoglobin [24, 26, 27, 29, 30], three provided iron biomarker measurements [21, 26, 29], one measured anthropometry, morbidity, risk of malaria and lower genital tract infections, haemoglobin and iron biomarkers [21], and one measured maternal mortality [28]. All the community-/population-based studies had no end assessment for participants who remained non-pregnant, except one study which measured iron deficiency anaemia [33]. Those without a non-pregnant evaluation were primarily assessing neural tube defect prevention and did not anticipate side effects with the use of folate supplements. Four studies reported neural tube defects alone [20, 22, 34, 36], five reported birth weight measurement and neural tube defects or birth defects [20, 22, 34, 36, 37], twelve included birth weight outcomes [21, 25, 26, 28–30, 33–38] and variably measured gestational age [21, 26, 29, 35, 37], neonatal anthropometry [25, 29, 37], and maternal and neonatal iodine status [38]. Only two trials included tissue placental sampling [21, 29] or placental function [27, 29]. Consent was a single process except for one study which took consent at each key stage: enrolment, first pregnancy visit and for a follow-up infant survey [21].

### Trial design

**Fig. 3:**
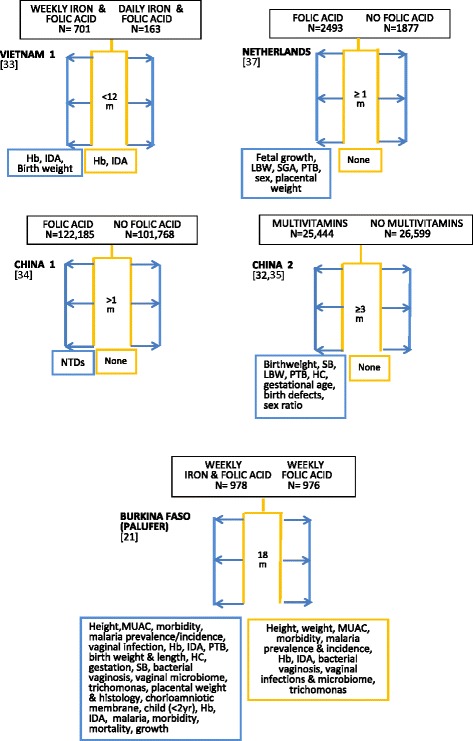
*(Continued)*

Figure 3 provides a structural outline for a periconceptional intervention trial using individual randomisation at enrolment to intervention or non-intervention arms. This template illustrates that four cohorts arise from a periconceptional design, two non-pregnant and two pregnant.

In terms of trial inclusion criteria, the pregnancy rate will be optimised if only women who have experienced menarche are selected. Exclusions at enrolment generally include currently pregnant women and those with significant illnesses. In low resource settings, moderately severe anaemia (Hb < 10 g/dl) may be a criterion for exclusion. Enrolment of anaemic women avoids selection bias but creates a heterogenous cohort, as those with nutritional anaemias may benefit more from supplementation than non-anaemic women, with different outcome effects. Women with anaemia observed at baseline would normally require treatment with iron and folate supplements. This might compromise study outcomes if large numbers had to be treated, especially in developing countries where two-thirds of women are typically anaemic. Treatment is sometimes reserved for severe anaemia, providing adequate follow-up of the less anaemic participants is possible. An alternative would be treating participants with clinical anaemia and without haemoglobin assessment. Sera collected at baseline could be stored for ethically approved retrospective measurement of nutritional biomarkers. Outcome assessments would then be based on differences in mean haemoglobin values at follow-up, precluding comparison of change from baseline haemoglobin concentration [39]. For the studies outlined in Fig. 2, eleven collected no baseline blood sample for haemoglobin [21–23, 25, 28, 30, 32, 34–37], and one indicated clinical screening for anaemia, with baseline sera storage, and exclusion only if hospital treatment was required [21]. Of the remaining studies, five measured baseline haemoglobin [24, 26, 29, 31, 33], two excluded participants if this was <7 g/dl [24, 26], one if <8 g/dl [29], one treated if <7 g/dl and re-recruited [31], and one did not describe study practice [33].

Comprehensive baseline assessment is central for assessing the magnitude of change in outcome measures [39]. Ideally this would include dietary studies, as the effect of the supplement has to be distinguished from a non-specific effect of diet [20]. Other confounding factors or biases such as maternal mental health or workload activities [26] may generate associations between exposure at baseline and the outcome and need to be pre-specified if anticipated, or indicated as post hoc exploratory analyses [40].

In terms of monitoring visits, Fig. 3 does not specify frequency for the non-pregnant and pregnant cohorts, as this is governed by the timing of supplement delivery (daily, weekly, monthly), method(s) of adherence assessment and the frequency of safety assessments. A weekly structure was employed in the PALUFER trial of weekly iron and folate supplementation because it addressed safety issues which required weekly follow-up visits [21]. The intensity of follow-up may itself influence adherence.

#### The non-pregnant cohort

In periconceptional studies, the trial design generally focusses on the pregnancy cohort and pregnancy-related primary outcomes. However, the evaluation of the non-pregnant cohort is important, as secondary outcomes, such as anaemia, specific nutritional deficiencies, or inflammatory or biomarker profiles can be measured and compared with the pregnant cohort. For studies including parous women, parity-related hypotheses can be evaluated such as effects of supplementation on fertility in different parity cohorts [41], growth in nulliparous adolescent women [19], or predictive value of nutritional parameters at baseline for outcomes at the end assessment [42]. This requires collecting relevant anthropometric and nutritional data.

Non-pregnant women may perceive less benefit from supplement use before pregnancy, and adherence levels in periconceptional trials may differ from pregnancy supplementation studies starting after the first trimester [24]. Follow-up of the non-pregnant cohort after discontinuation of supplementation may be indicated if latency in onset of adverse events might occur (for example, with delayed incidence of infection) or if pregnancy is probable soon after cessation. Monitoring unscheduled health visits is useful, as it may reflect adverse events or relate directly to trial outcomes.

Figure 3 includes an interim cross-sectional survey which can provide an interim safety analysis for trials with infection parameters as primary outcomes, or if there is a substantial concern about side effects. An interim survey needs to be carefully designed, especially if treatment is required which may influence outcomes. An interim survey may not be required if there is a long lag between recruitment and availability of the primary outcome at birth (for example, birth size) [29].

#### The pregnant cohort

Identification of conception can be based on a reported missed menstrual period with confirmation by a pregnancy test. Some pregnant participants might still be excluded, dependent on whether a minimal period of non-pregnant supplementation was pre-specified. Trial re-entry for participants with repeat pregnancy during the period of supplementation would normally be an exclusion criterion as the two pregnancies would not be independent, or if only nulliparous women were eligible.

In the majority of studies in this review, trial supplementation continued through early gestation to a scheduled first or early second trimester visit. For practical reasons this first scheduled study visit could correspond with the first visit for routine antenatal care when an ultrasound assessment can also confirm gestational age. This visit may be used as an end-point if parameters early in gestation are related to the primary outcome. After the first antenatal study assessment, and depending on study objectives, the intervention supplement may be stopped in order to allow the pregnancy cohorts in both arms to receive standard antenatal iron and folate supplementation, possibly in higher doses than in the intervention supplement, and following national guidelines. If study outcomes are measured at delivery, then re-consenting the individual to pregnancy follow-up should be considered as part of Good Clinical Practice (GCP), especially as there are two distinct cohorts (pregnant and non-pregnant) in the study design. The same applies to any further infant follow-up.

The frequency of study visits during pregnancy may vary according to biological or obstetric outcomes to be measured, although at least one further antenatal attendance prior to delivery would be appropriate for monitoring purposes. Assessment at 32–34 weeks gestation may be the most practical, as it provides near-delivery clinical indicators (for example, infection status, anaemia, blood pressure) which can be used as proxy measures if assessment at delivery may be missed, as is often the case with village deliveries in developing countries. Monitoring of unscheduled pregnancy visits may be required to pick up pregnancy complications or intervening treatments. Adherence to routine antenatal iron and folate supplements can be assessed from the antenatal card or using monthly tablet counts. Frequent monitoring may lead to study fatigue, which may be reduced by assistance with transport and free health care provision, providing these are not provided as active inducements.

At delivery, clinical outcomes are assessed as part of standard care, but because of the importance of placental assessment in periconceptional interventions, it is a priority to obtain adequate placental tissue, with chorioamniotic membrane samples. Processing for placental histology may have specific requirements, for example, malaria histopathology [43]. Placental weight should be measured after trimming of the cord and marginal membranes.

At delivery, duplicate anthropometric measurements reduce measurement error of infant length, which can be measured as crown-heel and crown-rump length, together with the baby’s head and abdominal circumferences. In addition to newborn examination, a clinical gestational age assessment is advantageous, especially if pregnancy ultrasound assessments are not routine. The time of cord clamping should be established, as this influences anaemia risk in infancy [44], although this might be difficult for babies delivered at home by traditional birth attendants. Post-partum outcomes would require a scheduled study visit in the first week, with an additional postnatal visit at 4–6 weeks.

#### The infant cohort

Only three of the seventeen periconceptional studies reviewed included infant follow-up assessments, two of which adopted a cohort design [23, 29], and one a cross-sectional survey assessment [21]. There is an issue about statistical power if these outcomes are being measured opportunistically, and in analysis, inadequate power reduces the impact of any negative findings. If powered, large cohorts may be required and multiple statistical comparisons will need to be addressed.

The importance of infant follow-up relates to predictive risk of infant micronutrient status on infection risk and assessment of potential benefits to the offspring [45]. The operational framework for infant follow-up should be established early in the study, as infants can be born within seven months of enrolment or sooner if participants are already pregnant at recruitment. Infant follow-up will require active household visits and/or linkage with routine postnatal care and child health care visits. Infant assessments at 6 weeks, 3, 6, and 9 months and at one year of age would be appropriate for screening for anaemia, vaccination status and infection profiles, with less frequent scheduling in the second year. The timing of these would vary according to the outcomes of interest, but an assessment survey at around 9–12 months of age would be suitable as infant haemoglobin would normally plateau by that time. This timing would facilitate initial developmental and auditory testing, infant feeding practices and assessment of core breast feeding indicators. An alternative, more pragmatic, but less informative approach for assessing infant outcomes would be to complete a single cross-sectional survey for all study infants after the last trial infant is born. The mean age of children in this survey would be expected to be comparable by trial arm, unless the intervention had influenced child survival.

### Generating mechanistic hypotheses as a trial basis

Mechanisms can be considered in a general classification related to maternal, placental or fetal factors. These will interface, and underlying hypotheses should consider this interaction. Information from human, animal or laboratory studies should be considered. Table 2 summarises putative mechanisms using the above classification.

**Table 2:** Maternal, placental and fetal mechanistic hypotheses

Maternal (biochemical, endocrine factors, and plasma volume)
- Glucocorticoid effects on the fetal hypothalamic-adrenal axis (see fetal effects) [54, 82]
- Glucose homeostasis on metabolic responses leading to greater fetal fat deposition, insulin secretion, DNA methylation, and differential development of fetal endocrine systems [51, 55]
- Specific mineral deficiencies and impaired metabolic pathways [82]
- Limited macronutrients required for fetal growth [82]
- Adiposity [83]
- Oxidative stress associated with deficiencies of specific micronutrient antioxidant activities [84]
- Infection with transcriptional inflammatory response through IL-6 receptor alpha and prostaglandin response leads to cervical ripening and uterine contractions [85]
- Homocysteine metabolic effects on obstetrical vascular disease or placental spiral artery function [86]
- Interferon tau and progesterone effects on selective nutrient transport to the uterine lumen, with cell signalling pathways effecting, migration, and protein synthesis in trophectoderm [64]
- Urinary metabolites measured at the end of the first trimester and increased risk of negative birth outcomes [87]
- Biochemical markers of early placentation and downstream resistance to uterine arterial flow [77]
- Sex-specific effects of first trimester progesterone levels [52]
Placental (growth, morphology, vascularisation and function)
- Placental oxygen consumption is greater than fetal, and anaemia and placental hypoxia lead to free radical production, alteration in placental size, and vascularisation [15, 88–90]
- Alterations of number and surface area of arterioles in tertiary villi, and factors controlling endothelial re-modelling and trophoblast cell turnover from immature villi to conductance villi and gas exchanging terminal villi [16]
- Impaired molecular signalling networks [91]
- Reduced transfer capacity due to impaired utero-placental flow and fetal nutrient uptake [92]
- Impairment of fetal trophoblast and angiogenesis due to oxidative stress and inflammation [15, 93, 94]
- Long-chain polyunsaturated fatty effects on placental weight and surface area of gas exchanging placental capillaries [89, 95]
- Iron associated with markers of vasculopathy and placental growth factor excess [96]
- Alterations in placental phenotype and availability of placental hormone receptors and effects of hormones on the morphology, transport capacity and endocrine function of the placenta [56]
- Decreased nitric oxide bioavailability through low dietary arginine substrate and antioxidant supply [82]
- Interference with folate homeostasis in malaria infected placentae [97]
Fetal (embryonic, fetal growth factors and endocrine axes)
- Periconceptional undernutrition accelerating fetal hypothalamic-pituitary-adrenal (HPA) axis activation [54], mediated by different influences of maternal glucocorticoid on maturation of the fetal HPA axis, via placental 11-beta-hydroxysteroid dehydrogenase isozymes. Fetal exposure to glucose, fatty acids, and micronutrients, with resultant increased fetal insulin secretion, could influence development of the HPA axis controlling infant appetite [57]
- Placental metabolic alterations associated with the growth restricted fetus [89]
- Pre-term birth frequency in the growth restricted fetus [98]
- Altered fragility of chorioamniotic membrane [99]
- Inadequate micronutrient supply [48]
- One-carbon metabolic effects on methyl groups and DNA methylation [100]
- Fetal epigenomic effects during early stages of embryogenesis leading to stable and inheritable alterations in genes through covalent modifications of DNA and gatekeeper genes leading to nutritional programming [53, 101]
- Transport effects on methionine from the mother to the coelomic cavity and amniotic fluid [64]
- Vitamin independent effect of homocysteine in the fetal metabolic cycle [64]
- Thyroid hormone effects of mild/moderate iodine deficiency on cognitive ability and growth [102]
- Fetal angiogenic and placental growth factors affecting newborn thyroid function [103]
- Association of rapidly growing fetus with increased vulnerability to impaired nutrient supply [104, 105]
- Influence on development and activation of regulatory T cells in the human fetus [106]

#### General mechanisms

These may not account for compensatory growth mechanisms, such as the placenta adapting by up-regulating its transfer systems, and some changes may be irreversible, such as altered expression of transcription factors which in turn produce reduced cell content and enzyme actrivity [46]. Cell cycle regulation and cytoskeletal remodelling are critical processes in the nutritional programming of embryonic development [47], and rapid fetal growth may increase vulnerability to impaired nutrient supply [48]. Even a minor variation in maternal nutritional status is capable of producing important shifts in the fetal environment, as demonstrated in animal studies [49]. There is also evidence that nutrient status in the immediate pre-conception period may affect fertility [41, 50].

#### Specific mechanisms

The possibility of sex differences in studies concerned with birth size should be considered because of sex differentials in growth [51], sex-specific effects of first trimester progesterone levels on female birth weight [52], and sex-specific epigenetic effects [53]. A number of nutrient- and endocrine-related mechanisms are listed in Table 2, including interfacing hormone receptors on the placenta and fetal-hypothalamic axis [51, 54–57]. Although all trials include surveillance for adverse events, there may be a basis for evaluating hypotheses related to adverse events [58]. An example would be when potential nutrient-infection interactions suggest negative outcomes for some participants. One periconceptional trial measured malaria risk in early gestation in participants randomised to receive weekly iron and folate supplements, or folate alone [21]. The hypothesis was that *Plasmodium falciparum* parasites utilise iron for growth [45, 59].

### Selection of single versus multimicronutrient interventions

While there is consensus on use of folic acid in the periconceptional period for prevention of neural tube defects, opinions vary on whether to use single micronutrient supplementation, or composite multimicronutrients including the nutrient of interest [10]. In low resource settings the rationale for multimicronutrient supplementation is influenced by high population prevalence of chronic undernutrition in pregnancy, and a broad nutritional approach is pragmatic. In such settings a single micronutrient supplement may be less likely to improve placental function and fetal growth. Conversely, in areas where overnutrition is common, the developmental origins of health and disease hypothesis suggests that fetal exposure to additional glucose, fatty acids and micronutrients may increase fetal insulin secretion, influencing the development of the hypothalamic-endocrine system, which controls appetite [60, 61].

The rationale for the composition of mineral and vitamin multimicronutrient supplements is often unclear. Individual nutrient dosage is often based on dietary recommendations using physiological requirements, but there are substantive differences between trials. Table 3 illustrates this variation in composition for the seventeen reviewed studies. Five used folic acid alone, and were designed to assess efficacy for prevention of neural tube defects in women with a previously affected infant [20, 22, 23, 25, 36]. Different supplement multimicronutrient content, dosages, and consumption patterns, with or without food, could influence effects. Uniformity across future periconceptional trials would facilitate comparative analyses.

**Table 3:** Nutrient content of vitamins and micronutrients used in supplementation trials outlined in Fig. 2

Trial country [reference]	Ca mg	Cu mg	Folic acid mg	I μg	Fe mg	Mg mg	Mn mg	Niacin mg	P mg	K mg	Se μg	Zn mg	Biotin μg	Vitamins									
														B1 mg	B2 mg	A μg	B12 μg	C mg	D IU	E mg	K μg	B5 mg	B6 mg
Wales [20]	-	-	4	-	-	-	-	-	-	-	-	-	-	-	-	-	-	-	-	-	-	-	-
Gambia [27, 31]	-	2	0.4	150	30	-	-	18	-	-	65	15	-	1.4	1.4	240	2.6	70	200	1	-	-	1.9
Hungary [30]	125	1	0.8	-	60	100	1	19	125	100	-	7.5	200	1.6	1.8	1,800	4	100	500	1	-	-	2.6
Bangladesh [24]	-	-	0.4	-	60	-	-	-	-	-	-	-	-	-	-	-	-	-	-	-	-	-	-
India [25]	240	-	4	-	120	-	-	15	-	-	-	10	-	2.5	2.5	1,200	-	40	400	-	-	-	2
Cuba [36]	-	-	5	-	-	-	-	-	-	-	-	-	-	-	-	-	-	-	-	-	-	-	-
Vietnam 1 [33]	-	-	3.5	-	60 ^a^	-	-	-	-	-	-	-	-	-	-	-	-	-	-	-	-	-	-
Netherlands [37]	-	-	0.4^b^																				
China 1 [34]	-	-	0.4	-	-	-	-	-	-	-	-	-	-	-	-	-	-	-	-	-	-	-	-
China 2 [32, 35] ^c^	100	2	0.4	-	10	30	3	14	77	4	30	10	100	1.4	1.4	169	3	60	200	8	-	4	-
Burkina Faso [21]	-	-	2.8	-	60	-	-	-	-	-	-	-	-	-	-	-	-	-	-	-	-	-	-
Ireland [22]	480	-	0.36	-	50	-	-	15	-	-	-	-	-	1.5	1.5	1,200	-	40	400	-	-	-	1
Algeria [38]	-	-	-	240	-	-	-	-	-	-	-	-	-	-	-	-	-	-	-	-	-	-	-
Multi-country 1 [23]	-	-	4	-	-	-	-	13	-	-	-	-	-	1.5	1.5	1,200	-	40	400	-	-	-	1
Multi-country 2 [29]^d^	280	4	0.4	250	20	65	2.6	36	190	200	130	15	-	2.8	2.8	800	5.2	100	1,000	20	45	7	3.8
Vietnam 2 ^c^ [26]	-	2	2.8	150	60	-	-	18	-	-	65	15	-	1.4	1.4	800	2	70	600	10	-	-	-
Nepal [28]	-	-	-	-	-	-	-	-	-	-	-	-	-	-	-	7,000	-	-	-	5	-	-	-

^a^Weekly dose of 60 mg increased to 120 mg week when pregnant. Trial compared weekly with a daily dose of 60 mg iron and 250 μg folic acid

^b^Folic acid 0.4 or 0.5 mg and 15 % used as part of a multivitamin supplement regimen

^c^In addition 100 μg molybdenum

^d^Nutrient content in lipid-based supplement containing: 118 kcal energy, 2.6 g protein, 10 g fat, 4.59 g linoleic acid, 0.59 g α-linolenic acid

Table 4 summarises considerations related to the selection of single or multimicronutrient interventions. In principle, this decision should relate to a mechanistic hypothesis, although in practice, holistic considerations may be prioritised, such as a high population prevalence of several micronutrient deficiencies. The selection of control groups can be problematic. Trial designs frequently use pregnant women who received iron and folic acid supplements as controls, although iron may be included in the composite supplement for the intervention group. Ethically this meets international recommendations for the prevention of pregnancy anaemia, but concomitantly restricts the facility to examine some nutrient-specific hypotheses. Supplements given to control, or placebo groups varied between those receiving two, three, five, or fewer micronutrients [6, 30].

**Table 4:** Factors related to use of a single or multimicronutrient periconceptional supplements

Single nutrient supplementation	Multimicronutrient supplementation
General points:	General points:
(a) Potential negative interactions between multiple nutrients [107] (b) Identification of the gestational timing of specific nutrient effects (c) Allows specific hypotheses to be tested as single nutrient effects can be identified, e.g., calcium supplementation and pre-eclampsia [108], or folic acid and neural tube defects (d) Preferred for assessment of dose–response associations (e) Facilitates safety and adverse outcome assessments, e.g., infection risk with iron supplementation (NIH), or folate use and cancer risk [109]	(a) Nutrient synergisms enhance potential benefits [110] (b) Theoretical need for multiple nutrients from early in gestation, and for normal placentation (c) Balanced supply of carbohydrates, lipids, proteins and vitamins is critical to meet fetal and maternal energy needs, and for substrates for metabolic pathways [104, 111] (d) Unlikely single nutrient intervention will improve placental function, as combined deficiencies are common in low resource settings, and combination of nutrients potentially ameliorates several underlying nutrient deficiencies (e) Requirement for ideal mixture of functional amino acids and micronutrients to regulate key metabolic pathways [82] (f) Optimal nutrition from early in pregnancy may help ameliorate need for advanced therapies of neonatal care in low resource settings (g) Greater increase in body stores of nutrients than with single nutrient supplementation
For iron:	Specific multimicronutrient effects:
(a) Targets pre-existing iron deficiency anaemia and addresses need to enter pregnancy with adequate iron stores [112] (b) Data from experimental animals that iron status early in gestation may effect auditory responsiveness [113] (c) Assessment of specific interactions related to safety, e.g., iron-infection interactions influencing susceptibility to infection [14]	(a) Vitamins B2, B6, B12, magnesium and iron combined with folic acid may have greater protective effect in reducing risk of neural tube defects [30, 35, 114] (b) Benefits in improving content of breast milk for several nutrients [115] (c) Nutrient-nutrient synergisms may enhance iron absorption [116] (d) Folate and other vitamins measured longitudinally in pregnancy have values mostly below recommended levels [64], and accelerated breakdown suggested in addition to haemodilution [117] (e) Observational studies for less growth restriction and reduced pre-term birth with regular periconceptional multivitamins [118] (f) Evidence for improved birth weight with later gestational supplementation [8]
For folate:	
(a) Specific maternal and fetal metabolic enzyme polymorphisms can be targeted (e.g., methyl tetrahydrofolate reductase) [64] (b) Folate requirements increase steeply once the chorioallantoic placenta is formed and the fetal heart starts perfusion (about 22 days after fertilisation) (c) Folate and vitamin B12 linked to utero-placental vascular resistance [119]	
For iodine:	
(a) Mild to moderate iodine deficiency may influence cognitive development [102]	
Placental and genetic	
(a) Specific nutrients may be involved in expression of genes involved in placental function and cell cycle processes [120] (b) Identification of factors controlling trophoblast turnover from immature to mature villi	

Brackets: reference number

### Cohort size and composition

Study sample size is governed by non-inferiority (or superiority) margins between trial arms, and estimates of numbers lost to follow-up, migrations, withdrawals, and deaths. These latter factors may be considerable for studies undertaken in low resource settings. The proportion of marriageable young women may affect the study sample, as marriage generally involves movement to a new home. Exclusion of participants who indicate an imminent change in residential location at enrolment is sometimes adopted, but such movements are not always predictable and the effect on loss to follow-up may be substantial. If the primary outcome is a pregnancy variable, and only a minority of the pre-pregnancy cohort subsequently conceive, the protective effect of randomisation against bias may be weakened.

A standard definition of fertility is the number of live births per 1,000 women aged 15–49 years in a given year (number of births per year/number of women 15–49 years). An estimate of the population fertility rate is essential in order to estimate accrual of pregnancies, the period of time to attain this, and the size of the residual non-pregnant cohort. In practice accurate fertility estimates are only available in areas where there has been a recent census, or if the study is undertaken in a demographic surveillance area. Approximate fertility estimates may be misleading and can jeopardise trial integrity if the approximation substantially overestimates the true rate.

As an example, Fig. 4 shows fertility rates, by five-year age groups and birth order, for Moroccan women using National Demographic Health Surveys (DHS) data [62]. Based on these fertility rates, parity and maternal age effects across groups would have an impact on cohort study design, because age-specific fertility depends on the proportion of the fertility distribution spent in each category. An average fertility rate would be confounded by residual parity and age effects. These may be considerable over a long period of trial supplementation. Using the DHS data plotted in Fig. 4, the estimated number of births by differential age and parity fertility rates (parities 1, 2, and 3), and for increasing population sample sizes, are shown in Table 5. This illustrates the substantial numerical variation between class groups, emphasising the importance of accurate demographic data for estimating the sample size. This will be dictated by whether study objectives relate to all parity groups or focus on a specific group such as nulliparae. An example was the PALUFER periconceptional trial, in which malaria in primigravidae early in pregnancy was the primary outcome, as malaria affects more primigravidae than multigravidae [21].

**Fig. 2:**
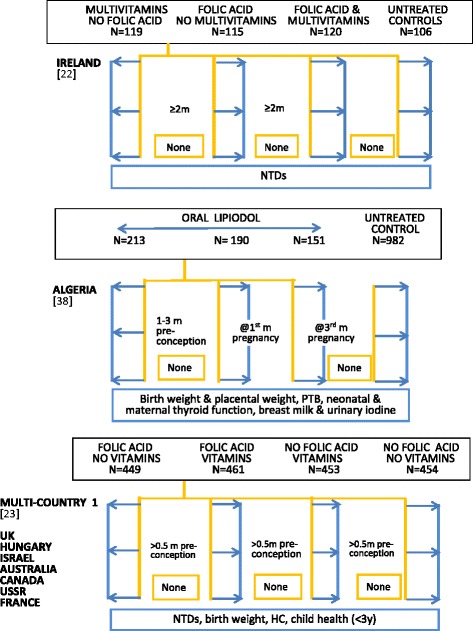
*(Continued)*

**Table 5:** Estimated annual numbers of births by differential age and parity (P1, P2, P3) fertility rates

Population of women of child-bearing age (n) ^a^	15 – 19 yrs			20 – 24 yrs			25 – 29 yrs			30 – 34 yrs			35 – 40 yrs		
	Fertility rates			Fertility rates			Fertility rates			Fertility rates			Fertility rates		
	P1	P2	P3	P1	P2	P3	P1	P2	P3	P1	P2	P3	P1	P2	P3
	54.6	23.5	8.2	76.2	75.4	53.0	37.2	52.7	64.8	15.0	24.5	34.5	3.2	5.8	14.8
1,000	55	24	8	76	75	53	37	53	65	15	24	35	3	6	15
2,000	110	48	16	152	150	106	74	106	130	30	48	70	6	12	30
3,000	165	72	24	228	225	159	111	159	195	45	72	105	9	18	45
4,000	220	96	32	304	300	212	148	212	260	60	120	140	12	24	60
5,000	275	120	40	380	375	265	185	265	325	75	144	175	15	30	75

^a^Demographic population size

If the study allows recruitment across parities, prior construction of a differential age-parity fertility rate table will be helpful in estimating the sample size. This can be done using DHS, recent census or local demographic surveillance data, thus avoiding inaccurate approximations that may require extension of the enrolment period. One periconceptional trial in this review was discontinued because of low fertility rates, combined with a fall in occurrence of the primary outcome of neural tube defects [22]. Secular declines in fertility, particularly in Asia, may occur between censuses and need to be allowed for. Sub-Saharan Africa is the only major region in the developing world that has not yet undergone a general decline in fertility.

Figure 5 shows the increase in the cohort sample size of pregnant women, and proportional decrease for the non-pregnant cohort, using data from Table 5. This is plotted for six-monthly sequential supplementation periods for a total of 18 months supplementation, and using an enrolment sample of 2,000 nulliparous women. It highlights a number of points: with higher fertility rates shorter periods of supplementation are required to reach an expected sample size; lower fertility requires longer supplementation periods for non-pregnant women; the non-pregnant cohort experiences longer periods of supplement exposure than the pregnant cohort for all fertility rates; if the duration of supplementation is an important variable (for example, for safety assessments, tolerability), then the sample size should be estimated according to this variable; there are programmatic implications, as the impact of different durations of supplement delivery relate to the fertility rate.

**Fig. 2:**
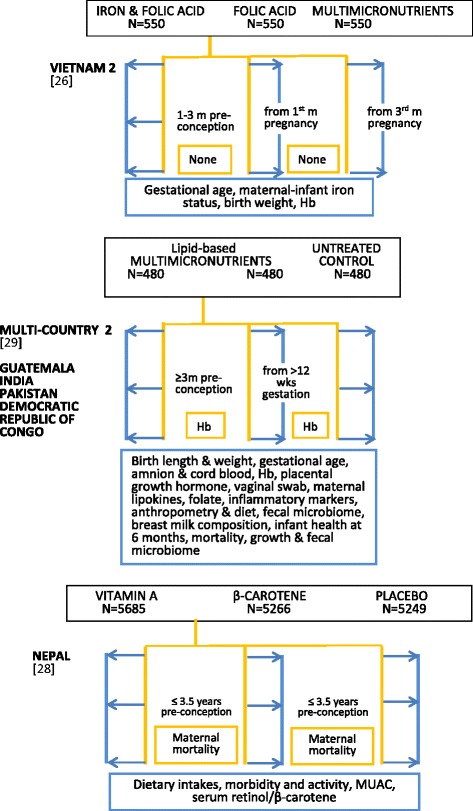
*(Continued)*

### Outcome measurements

Assessment options will be dictated by study hypotheses, although a broad-based approach to profiling outcomes is useful. Table 6 summarises the range of parameters to be considered for non-pregnant and pregnant cohorts, for post-partum and infant assessments, and biochemical markers relevant for placental studies. These are grouped by categories related to study activities and potentially relevant mechanistic hypotheses. Relevant specimen sampling is described which covers major biological options to be considered. In view of epigenetic phenomena and the potential associations between maternal micronutrient status and genetic variants in metabolic enzymes affecting health, genotype profiling is included [63]. The seminal example is reduced efficacy of periconceptional folic acid supplementation with deficiency of folate metabolising enzymes [64], but a much broader approach assessing multiple enzyme loci is possible [65]. Ethical permission for collection and storage of blood spots and sera should be anticipated if the study aims do not relate to a primary genetic hypothesis, as it would permit post hoc genotype profiling. This is especially relevant if study protocols are complex, prolonged or costly, as repeat studies may not be feasible and future studies would be potentially biased by time-varying confounding.

**Table 6:** Assessment considerations in periconceptional supplementation trials

Study activity	Non-pregnant	Pregnant	Placenta	Post-partum	Child (birth – 24 months)
Health history and assessment	Exclusion criteria^a^ Demographic and socio-economic status; Food security, dietary and drug history; Morbidity, obstetric and reproductive histories; BP; mental health; workload	Uterine artery pulsatility (UtAPI) and resistance indices (UtARI) at 28–32 w gestation;^b^ BP; Ultrasound: 1^st^ trimester: gestational and yolk sac development; 2^nd^/3^rd^: biparietal diameter, HC, AC, FL; LMP; drug use	History of pre-eclampsia	Mental health	Gestational assessment, infant feeding practices, neuro-behavioural assessments, fat-free mass oto-acoustic emissions; drug use
Anthropometry ^c^	Wt, Ht, MUAC, Skin-fold thickness, BMI, ultrasound of abdominal visceral fat	Wt, Ht, MUAC	Wt, diameter	Wt, Ht, MUAC, BMI	Wt, recumbent Lt, crown-rump Lt; head, upper arm, abdominal circumference
Haematology	Baseline anaemia	Hb, MCHC, MCV, red cell distribution width	Cord Hb, ferritin, sTfR	Hb, cord clamping time	Hb at 3, 6 12 months
Biochemistry	Iron biomarkers,^d^ sera and RBC folate, micronutrient profiling, metabolome	Iron biomarkers, sera and RBC folate; amino acids, lipids, fatty acids, renal function, gluconeogenesis, anti-oxidant profile,^e^ metabolome	Ratio plasminogen-activator inhibitor (PAI)-1: PAI-2,^f^ uterine-artery Doppler waveform at 18–22 weeks gestation,^f^ cord blood metabolites^g^	Iron biomarkers, micronutrients, amino acids, lipids, fatty acids, breast milk composition	Iron and folate biomarkers, amino acids, lipids, fatty acids, micronutrients, anti-oxidant profile,^e^ metabolome
Endocrinology	Adipose tissue-secreted hormones (adipokines): leptin, visfatin, resistin, apelin, omentin, sex steroids, growth factors	Cortisol, progesterone, oestradiol, thyroid function, pregnancy associated plasma protein-A (PAPP-A); free β-human chorionic gonadotrophin (β-hCG)	Cord placental growth factor (PlGF); soluble FMS-like tyrosine kinase-1 (sFlt1)^h^	Adipokines	Hormonal growth factors, glucose homeostasis
Infection	Blood/stool samples, STIs^i^ HIV, bacterial vaginosis, vaginal microbiome	Bacterial vaginosis, STIs	Chorioamnionitis, malaria histology	Bacterial vaginosis, vaginal microbiome	Gut helminths, malaria, respiratory, diarrhoea, HIV, health attendances, fecal microbiome, thymic size
Inflammation	CRP, AGP	CRP, AGP	Specific maternal-cord antibody titres	CRP, AGP	CRP, AGP
Genotype profile	Blood storage	-	Micro RNAs^j^	-	Blood storage, micro RNAs

*Abbreviations: Wt* weight, *Ht* height, *MUAC* mid-upper arm circumference, *BMI* body mass index, *HC* head circumference, *AC* abdominal circumference, *FL* femur length, *CRP* serum C-reactive protein, *AGP* alpha-1-acid glycoprotein, *Hb* haemoglobin, *BP* blood pressure, *STI* sexually transmitted infections, *LMP* last menstrual period

^a^ Dependent on study design and location these would include: sickle cell disease and hemoglobinopathies; severe anaemia (Hb <7 g/dl); diabetes; current pregnancy; pre-menarcheal subjects; severe malnutrition or other severe illness (see study design section)

^b^ The uterine artery pulsatility index (UtAPI) and resistance index (UtARI) at 28–32 weeks gestation and record of diastolic notching. Quantify systolic and diastolic components of the flow velocity waveform in a specific blood vessel over a single cardiac cycle, with higher values indicating downstream vascular resistance

^c^ Duplicate measurements

^d^ Serum ferritin, transferrin receptor (sTfR), hepcidin, free erythrocyte protoporphyrin, transferrin saturation

^e^ Metabolomic profiling

^f^ A surrogate marker of placental perfusion which correlates with trophoblast invasion [15]

^g^ Blood metabolites including: insulin, glucose, liver enzymes, amino acids, fatty acids

^h^ Placental growth factor (PlGF) is a proangiogenic factor sharing high homology with vascular endothelial growth factor; soluble FMS-like tyrosine kinase-1 (sFlt1) is a potent antagonist of vascular endothelial growth factor and PlGF signalling

^i^ Screening for regionally specific infections; parasitic infections (e.g., malaria, enteric helminthiasis, schistosomiasis); genital tract infections (e.g., bacterial vaginosis, STI syndromes)

^j^ Placental micro RNA expression of small non-coding RNAs that are involved in post-transcriptional gene regulation

Randomised trials are the best design for testing intervention effectiveness, but more detailed laboratory science is required to explore possible causal mechanisms that can inform the development of interventions. Profiling a wider biochemical range of nutrients with maternal, neonatal and infant outcomes could help to identify additional interventions. Advances in metabolomics now make this possible [66], with screening of large sample numbers using mass spectrometry or nuclear magnetic resonance spectroscopy. Novel biomarkers may be assessed for predictive accuracy for fetal growth restriction and other pregnancy outcomes [67, 68]. For these reasons a storage sera set should be secured for future examination, and potentially to allow cross-cohort comparisons for replication across studies and use of triangulation methods [17].

Some of the general profiling options outlined in Table 6 may not be measured for pragmatic reasons, or because they do not relate to study objectives. In order to illustrate a specific trial outline, Table 7 shows the activities for the PALUFER trial of malaria risk prior to and during early pregnancy in nulliparous women receiving long-term weekly iron and folic acid supplementation [21]. This was a non-inferiority randomised controlled trial based on infection risk parameters, where the primary endpoint was malaria infection at the first scheduled antenatal visit. Malaria prevalence in the non-pregnant cohort was also assessed at an end assessment survey. The study used the structure outlined in Fig. 3, which provided a platform for a series of secondary analyses, for pregnant and non-pregnant cohorts. Risk of bacterial vaginosis, a major risk factor for chorioamnionitis, was also assessed in both cohorts [69]. Detailed baseline profiling allowed the predictive value of biomarkers to be assessed for subsequent health outcomes. The large size of the residual non-pregnant cohort provided adequate statistical power for secondary outcome assessments. In the PALUFER trial, participants were consented at enrolment, re-consented at first antenatal visit, and again for the infant cross-sectional survey, and this included permission for sample storage.

**Table 7:** Profile of PALUFER trial activities evaluating weekly iron supplementation [21]

Study contact	Health history and assessment	Haematology	Biochemistry	Anthropometry	Infection and inflammation screen^a^
Screening	Demographics^b^	-	-	-	-
Randomisation	Reproductive,^c^ general health, blood pressure, temperature, dietary,^e^ drugs	Sera for iron biomarkers,^d^ Hb if pallor or symptomatic	Blood for sera and genotype	Wt, Ht, BMI, MUAC	Malaria if symptomatic, vaginal, STI (syndromic)
*Non-pregnant cohort*					
- Weekly visits^f^	T^0^ C and morbidity,^g^ side effects, compliance,	-	-	-	RDT for malaria if symptoms/febrile
- Cross-sectional survey	T^0^ C and morbidity, side effects	-	-	-	All for malaria microscopy
- Participant unscheduled visits	T^0^ C and morbidity, side effects	-	-	-	If symptoms
- End assessment survey	T^0^ C and morbidity, side effects	Hb, iron biomarkers	Nutritional biomarkers^h^	Wt, Ht, MUAC	Malaria RDT and microscopy, BV, CRP, vaginal microbiome and lactoferrin, trichomonas
-Focus groups/interviews	Knowledge and acceptability	-	-	-	-
*Pregnant cohort*					
- First AN attendance ^i^ at 13–16 wks	T^0^ C and morbidity, ultrasound, blood pressure, drugs (IPTp), supplement, compliance, side effects	Hb, iron and folate biomarkers	Urine glucose and protein	Wt, Ht, MUAC	Malaria RDT and microscopy, CRP and AGP, BV, HIV, STI (syndromic), vaginal lactoferrin and microbiome, trichomonas
- Second AN attendance at 32–36 wks	T^0^ C and morbidity, blood pressure, drugs (IPTp)	Hb if pallor or symptomatic	Urine glucose and protein	Wt, Ht	Vaginal lactoferrin and microbiome, other infections if symptoms
- Unscheduled visits	T^0^ C and morbidity	Hb if pallor or symptomatic	-	Wt	Other infections if symptoms
- Delivery	T^0^ C and morbidity, stillbirths	-	-	Wt	Placental histology for chorioamnionitis and malaria
*Infant follow-up*					
-Live births	Gestational age	-	-	Wt, length, HC	-
-Cross-sectional postnatal survey	Infant feeding, morbidity, health visits	Hb, iron biomarkers	-	Wt, length, MUAC	Malaria

*Abbreviations: Ht* height, *Wt* weight, *HC* head circumference, *BMI* body mass index, *MUAC* mid-uper arm circumference, *T* ^*0*^ temperature, *BV* bacterial vaginosis, *STI* sexually transmitted infection, *IPTp* intermittent preventive treatment for malaria (sulfadoxine-pyrimethamine), *HIV* human immunodeficiency virus, *CRP* C-reactive protein, *AGP* acyl glycol-protein, *Hb* haemoglobin, *RDT* rapid diagnostic test for malaria

^a^ Malaria, or other exposures, e.g., vaginal infections including bacterial vaginosis and trichomonas, HIV infection

^b^ Location, marital status, occupation, education, ethnicity, likelihood of migration, socio-economic status

^c^ History of menarche, sexual history, previous pregnancies, live births, stillbirths, sickle cell disease

^d^ Serum ferritin, transferrin receptor, hepcidin, free erythrocyte protoporphyrin, mean corpuscular Hb concentration (MCHC), red cell distribution width

^e^ Including use of nutrition supplements

^f^ Variable frequency dependent on study requirements (weekly supplements for PALUFER trial)

^g^ Questions related to fever, respiratory and gastro-intestinal symptoms, skin rashes, or since previous visit. Includes mortality record

^h^ Sera for vitamin and micronutrient concentrations

^i^ Gestational timing and frequency dependent on study objectives

### Adherence assessment

Assessment of adherence to supplementation in the periconceptional trials shown in Fig. 2 was generally by tablet counts, and reported as the percentage compliance. This is probably not sufficient, as percentage compliance obtained by counting tablets can be calculated in different ways. Trial details are shown in Table 8 with assessment frequency varying from weekly to every three months. One of these trials reported an outcome assessment (change in haemoglobin) by quartiles of percent adherence [24]. The epidemiology of supplement intake patterns requires better description than total tablet counts, and current trials should be able to provide a more detailed analysis of the potential influence of adherence patterns on study outcomes [21]. With longer supplementation adherence may be discontiguous, with intake discontinued and re-commenced for variable monthly periods, resulting in intermittent patterns and variable adherence exposure at the time of conception, especially as seasonal factors may be influential. Percentage compliance does not capture this variation, as in longitudinal trials adherence is a rate rather than a period prevalence, and would be more precisely expressed as a rate in person weeks/months of follow-up. One study used directly observed intake [21], which is a definitive method of assessment, but even this does not secure intake if participants are absent for variable periods. Trials need to be adequately powered to allow for this influence, in particular with long periods of supplementation.

**Table 8:** Supplement compliance and uptake for studies outlined in Fig. 2

Trial location (reference)	Double blind	Tablet regimen	Supplement duration	Adherence assessment method	Side effects monitored	Adherence
Wales [20]	Yes	Daily	Not reported ^a^	Serum folate cut-off	Yes	73 % Not assessed in controls
Gambia [27, 31]	Yes	Daily	Mean 10.9 weeks pre-conception to 11 weeks post-conception	Two weekly home tablet counts	Yes	88 %.
			Median 24.1 weeks			Supplement clinic attendance 72 %
Hungary [30]	No	Daily	Up to 9 months pre-conception to 3 months gestation	Three monthly tablet counts	Yes	71.5 % full course
						8.9 % no supplements^b^
						19.6 % partial supplements^c^
Bangladesh [24]	Yes	Daily	Maximum 9 months	Monthly sachet counts	Yes	57.7 ± 26.9 %^d^
			Mean 72.9 days			
India [25]	Yes	Daily	≥1 month pre-conception – 3 months post-conception	Three monthly tablet counts	Yes	34 % lost to follow-up before conception
Cuba [36]	No	Daily	One menstrual period before conception − 10 weeks gestation	Not reported	No	19.8 % partial supplements^c^
						55.1 % no supplements^b^
Vietnam 1 [33]	No	Weekly vs daily	Up to 9 months	Tablet purchases	No?	50-92.5 %, variable with period of follow-up
Netherlands [37]	No	Daily	Not reported	Self-reported	No?	29.6 % reported not using
China 1 [34]	No	Daily	Maximum 38 months	Monthly bottle counts	No	81–87 % periconceptional^e^
						74–75 % late use^f^
						68–78 % discontinued^g^
China 2 [32, 35]	No	Daily	At least 3 months pre-conception. Mean 149.8 days pre- and 49.3 days post-conception	Monthly capsule counts	No	85.7 % – 93 % compliance
Burkina Faso [21]	Yes	Weekly	Maximum 18 months	Directly observed intake	Yes	Trial in progress
Ireland [22]	Yes	Daily	At least 2 months pre-conception	Tablet counts and blood tests^h^	Yes	Not reported
Algeria [38]	No	Single dosage	Either 1–3 months pre-conception, or 1–3 months gestation	Directly observed	Not applicable	100 %^i^
Multi-country 1 [23]	Yes	Daily	Continuous until 12^th^ week gestation	Three monthly capsule counts	Yes	7 % discontinued;^g^ 3–8 % took 50–79 %; 0.8 % took <50 %
Multi-country 2 [29]	No	Daily	Not reported	Self-reported sachet use	Yes	Trial in progress
Vietnam 2 [26]	Yes	Daily	Maximum 18 months	Two weekly capsule counts	Yes	Trial in progress
Nepal [28]	Yes	Weekly	≤3.5 years	Directly observed	No?	>75 % pregnant ≥ 50 %
						62 % non-pregnant ≥ 50 %

^a^From time contraception stopped

^b^Zero adherence

^c^Incomplete adherence

^d^Percentage total eligible doses consumed

^e^Started supplement before last menstrual period before conception and stopped at end of first trimester

^f^Started supplement during first trimester but after last menstrual period

^g^Started and stopped supplement before last menstrual period before conception

^h^Count frequency not reported

^i^Assuming no refusals

The adherence level is one criterion used to distinguish per protocol from intention-to-treat populations. Of the studies outlined in Fig. 2, all used an intention-to-treat analysis approach without attempting to define a theoretical per protocol population. This definition would require a percentage compliance cut-off, or particular compliance profiles to be selected, the biological basis of which may be uncertain. This is particularly challenging where participants have highly variable uptake patterns and excellent adherence is impossible to achieve. With improved biochemical measures of exposure it may be possible to develop alternative assessment methods.

There are reasons to believe that adherence to medication may differ between arms, for example, if side effects varied by trial arm, or if the control group knew they were taking placebo, and so would not be independent of randomisation. Strata generated by adherence may therefore compare dissimilar patients from each treatment arm. There are therefore some problems in interpreting analyses that include a stratified adherence variable. It is nevertheless important to describe the level of adherence by treatment arm in a non-inferiority trial, as non-adherence results in a bias towards the alternative hypothesis. With non-compliance in the intervention arm, an adherence-based analysis may provide meaningful information (causal effect estimate of the treatment effect) [70]. The effect of non-adherence should be considered in secondary analyses.

### The role of qualitative studies

Only one trial in Burkina Faso included a qualitative component [21], in which focus group discussions with non-trial participants and field workers were conducted early in the study before supplementation had been established. The data drew attention to misconceptions about the purpose of supplementing unmarried, non-pregnant women in a setting in a developing country, which the team tried to address in subsequent community contacts (71). A high level of illiteracy in the rural study area compounded such misconceptions. Detailed interviews with participants who had varying levels of supplement adherence were also conducted at later stages, giving insights into unexpected factors that interfered with regular adherence and loss to follow-up, some of which could be addressed by the field team. Qualitative studies conducted in parallel with the main trial could assist in informing trial management, but may only be able to address a limited number of potential problems, especially in rural, more isolated communities in low resource settings. Examples include: falling adherence with longer supplementation periods; hidden pregnancies or abortions; adolescent pregnancies in unmarried women; miscarriages which may be interpreted as irregular menstruation, and their distinction from false positive pregnancy tests; misinformation on the nature of the intervention [71] or confusion with contraception; insufficient communication with local communities and partners or husbands. Some trials have enrolled only married participants, which may reduce occurrence of some of these factors, although in areas where unmarried adolescent pregnancy is common, this may result in selection bias. Protocol amendments may be required to address specific factors in order to ensure optimal trial participation, participant security, and community compliance. The Gambia trial did not integrate a qualitative component because it was a mechanistic study and also to avoid raising concerns about the nutritional supplement [72], and this restricted its ability to manage emerging issues. A study among non-participants illustrated extreme reluctance by women to disclose pregnancy in the first trimester.

The role of qualitative studies within trials has been diminished by views that such studies are relevant only for programmes, given that trials try to maximise adherence by intensive supervision and benefits (such as treatment and travel costs) that are not available routinely. Indeed, a community mobilisation and social marketing study to promote weekly pre-pregnancy iron and folic acid supplementation to married, nulliparous women in Vietnam reported that the purchase of supplements over four months fell from 92.5 % to 67.4 % [33]. In Burkina Faso the qualitative research has clearly shown that young women see little benefit from taking supplements and without free treatment, adherence would be much lower [21]. It would be better to anticipate such problems rather than have to initiate new studies to promote uptake following policy recommendations for supplementation.

### Data management

The data management of periconceptional trials can incorporate three categories of participants: non-pregnant, pregnant, and child cohorts, as well as longitudinal and cross-sectional data sets. All data can be handled in an electronic case record form (CRF) developed in MACRO (Infermed©). This is a powerful, industry-standard electronic data capture solution, which supports trials from Phase I through IV. It is designed to increase compliance with the requirements of relevant regulatory bodies including the International Conference on Harmonisation (ICH) Good Clinical Practice, and Federal Drug Administration-CFR21Part11 compliance. It also allows exchange of an entire clinical study between two different and independent study software solutions, facilitating exchange of metadata [73]. Considering the sample size and the long weekly follow-up period, source data collection of the weekly household visits using hand-held portable devices is preferable to facilitate real-time data validation, to avoid duplicate data entry, and to simplify batch data entry of large inter-related data sets.

The complexity of work flow with periconceptional trials requires an ability to effectively track participant compliance with complex protocol schemas (such as intervention schedules, data collection requirements, parallel cohort tracking, monitoring of side effects, sequential delivery times and participant recruitment, and participant out migration) [74]. Participant attrition as a result of protocol noncompliance can result in increased costs, delayed completion times, biased data, and ambiguity over protocol violations. Validated methods for clinical trial participant tracking are required [75], which will enhance data safety and monitoring reporting.

## Conclusions

There are many challenges facing early-life intervention studies, and trials in developing countries will have operational requirements which are context specific, and likely to be dependent on the literacy rate in the study population. This review has focussed on pragmatic issues that we hope can be drawn upon and expanded as experience grows with more completed studies. Periconceptional trials are distinguishable from pregnancy supplementation trials, not only because of the early gestational timing of nutrient exposure, but also because they generate a subsidiary trial in participants who remain non-pregnant. This provides an opportunity to evaluate concurrent hypotheses related to the health of women who may become pregnant. An expanded framework for conceptualisation, design, and implementation is required in order to reduce risks in achieving outputs, strengthen trial integrity, facilitate replication and data exchange and reduce costs. Basing trial design on a mechanistic hypothesis is an important pre-requisite which relates to the choice of a single or multiple micronutrient intervention. Micronutrient interventions are relevant across the entirety of a women’s reproductive period, even more so in young nulliparae and grand multiparae [76]. Longitudinal reference values for biochemical markers are required in uncomplicated pregnancies to facilitate interpretation [77]. Iron would be a requirement of most periconceptional supplements, especially in developing countries, as many women enter pregnancy with inadequate iron stores [78]. Early pregnancy iron deficiency has been associated with poorer [79], and iron supplementation with better birth weight outcomes [80, 81], although questions remain concerning iron-infection interactions which may have an impact on safety under specific infection exposures [14]. Folate is a requirement in view of strong evidence for periconceptional use preventing neural tube defects. The rationale for multimicronutrient dosage and content needs to be clearly outlined, in order to establish a comparative framework, improve standardisation, and facilitate interpretation of mechanistic hypotheses.

Other issues identified include: rationalisation of the duration of supplementation; problems with excluding anaemic participants at baseline; influence of follow-up intensity on intervention adherence; and out-migration, particularly in adolescent cohorts. Research and practice recommendations include: utilising accurate fertility data for both sample size and supplement duration estimations; prioritising placental tissue sampling; establishing appropriate safety assessments incorporating infection risk; inclusion of an infant follow-up; utilisation of electronic case record forms on MACRO (Infermed); and qualitative concurrent assessments prior to and during trial implementation. Qualitative assessments are not solely theoretical, as trial results will not appeal to policy makers if they are not implementable. This is a particular issue with pre-pregnancy studies and in first pregnancies, as women unfamiliar with routine care may fail to comply with supplementation. This may re-direct efforts towards food fortification, which are less realistic in some low resource settings. With more comprehensive assessments the complexity of the work flow and processes executed by the field clinical investigator should not be underestimated.

The heterogeneity of studies included in this review, which covers both randomised trials and prospective cohort studies, limited comparability. There was a predominance of studies examining NTD outcomes following folate supplementation, and considerable variation in duration of supplementation across studies. These differences related partly to assessment of alternative study outcomes, but most were not rationalised in physiological terms. While this lack of uniformity limited comparability, it facilitated identification of methodological issues relevant to future trial conduct.

## Abbreviations

AC: arm circumference
AGP: alpha-1-acid glycoprotein, acyl glycol-protein
AN: antenatal
BP: blood pressure
β-hCG: beta human chorionic gonadotrophin
BMI: body mass index
BV: bacterial vaginosis
Ca: calcium
Cu: copper
CRP: C-reactive protein
DHS: Demographic Health Surveys
DNA: deoxyribonucleic acid
FE: iron
FL: femur length
GCP: Good Clinical Practice
Hb: haemoglobin
HC: head circumference
HIV: human immunodeficiency virus
HPA: hypothalamic-pituitary-adrenal axis
Ht: height
I: iodine
IDA: iron deficiency anaemia
IPT_p_: intermittent preventive treatment with sulfadoxine-pyrimethamine
K: potassium
LBW: low birth weight
LMP: last menstrual period
Lt: length
LTFU: loss to follow-up
MCHC: mean corpuscular haemoglobin concentration
MCV: mean corpuscular volume
Mg: magnesium
Mn: manganese
MUAC: mid-upper arm circumference
NIH: National Institutes of Health (USA)
NTD: neural tube defects
P: phosphate
P1: P2, P3, parity one, two, or three
PAI: plasminogen activator inhibitor
PAPP-A: plasma protein A
PLGF: placental growth factor
PTB: pre-term birth
RBC: red blood cell
RNA: ribonucleic acid
SB: stillbirth
Se: selenium
STI: sexually transmitted infection
sTfR: serum transferrin receptor
T^0^: temperature
UtAPI: uterine pulsatility index
UtARI: uterine artery resistance indices
WIFS: weekly iron and folic acid supplementation
